# Adsorption Behaviors of ctDNA and Biological Activities Based on Polyvinyl Alcohol/Polyethylene Glycol/Quaternized Chitosan Composite Hydrogel

**DOI:** 10.3390/molecules29235770

**Published:** 2024-12-06

**Authors:** Lili Fu, Kun Liu, Jinyu Yang, Yuan Zhao, Zhijun Wang, Dongxu Tang, Yuesheng Li, Huangqin Chen

**Affiliations:** 1 School of Nuclear Technology and Chemistry & Biology/Hubei Key Laboratory of Radiation Chemistry and Functional Materials, Hubei University of Science and Technology, Xianning 437100, China; fll151852538462022@163.com (L.F.); 17754119632@163.com (K.L.); 18727791310@163.com (J.Y.); zhyf308@hbust.edu.cn (Y.Z.); qwertasdkl@163.com (Z.W.); tdtd800@126.com (D.T.); 2 School of Stomatology and Optometry, Hubei University of Science and Technology, Xianning 437100, China

**Keywords:** PVA/PEG/HACC hydrogel, ctDNA, radiation preparation, adsorption, bioactivity

## Abstract

In this study, a polyvinyl alcohol/polyethylene glycol/hydroxypropyltrimethyl ammonium chloride chitosan (PVA/PEG/HACC) ternary composite hydrogel was synthesized using electron-beam radiation. The materials were thoroughly characterized via Fourier transform infrared spectroscopy, X-ray diffraction, scanning electron microscopy, thermogravimetric analysis, Brunauer–Emmett–Teller analysis, gelation fraction tests, and swelling rate tests. Systematic adsorption experiments revealed that the rate of adsorption of calf thymus DNA (ctDNA) by the PVA/PEG/HACC hydrogel reached 89%. The adsorption process followed the Langmuir isotherm and pseudo-second-order kinetic model. This process was mainly characterized by monolayer chemical adsorption, with intraparticle diffusion playing a crucial role. In addition, the process was spontaneous, with higher temperatures enhancing adsorption. The possible adsorption mechanisms included electrostatic interactions, hydrogen bonding, and van der Waals forces. The maximum ctDNA desorption rate was 81.67%. The adsorption rate remained at 71.39% after five adsorption–desorption cycles. The bioactivity of the PVA/PEG/HACC hydrogel was validated by antibacterial, cytotoxicity, and apoptosis tests. The results of this study demonstrated the crucial application potential of adsorbent materials in DNA adsorption and biomedical applications.

## 1. Introduction

With the advancement of medical and health services, calf thymus DNA (ctDNA) has emerged as a crucial biomarker in liquid biopsy. It has played an important role in tumor research and clinical practice [1,2,3]. However, due to the short half-life and easy degradation of ctDNA in the blood, its extraction and enrichment process has been challenging. In order to meet this challenge, hydrogel adsorption technology can be used to extract ctDNA samples quickly and efficiently [4,5,6]. Therefore, the development of high-performance adsorption materials is crucial to overcome this difficulty, providing a reliable solution for the effective extraction and enrichment of ctDNA samples [7,8,9].

In early studies, common methods for ctDNA extraction and separation included phenol–chloroform extraction, chitosan magnetic beads, and column chromatography [10,11,12]. Janabi et al. [13] utilized phenol–chloroform extraction to isolate DNA from horse feces, demonstrating that this method can extract high-quality DNA samples suitable for processing large volumes of samples. However, this method was complex, time-consuming, and required significant amounts of chemical reagents. Alternatively, Shi et al. [14] used chitosan magnetic beads to extract DNA from trace amounts of cells. This method was simple and highly specific, allowing efficient capture of ctDNA, although its capture efficiency could be unstable. Chakka et al. [15] employed column chromatography for DNA extraction, which was known for its high extraction efficiency and broad applicability. Nevertheless, this method was time-consuming, costly, and lacked standardized procedures. Therefore, researchers have begun to explore different material adsorption techniques to improve the DNA extraction process. Hydrogel adsorption technology has garnered attention due to its large specific surface area, porous structure, tunability, low cost, renewability, and high selectivity. These advantages endow hydrogels with unique adsorption properties, making them a focal point in bioseparation and purification research. By fully leveraging the unique properties of hydrogels, researchers have designed and optimized adsorbent materials to enhance their separation and purification efficiency, thereby expanding the applications of adsorption technology in the biomedical and environmental fields [16,17].

Intelligent hydrogels have the advantages of intelligent regulation and can achieve intelligent changes according to external conditions or specific signals. In the field of adsorption, the responsive properties of hydrogels are used to achieve efficient adsorption and release of substances [18]. Li et al. [19] demonstrated the feasibility of utilizing smart hydrogels for DNA adsorption and sustained release. Researchers achieved efficient adsorption and precise controlled release of DNA molecules by surface modification and pore size adjustment of hydrogel materials. This provided a potential new adsorption material for biomedical and biotechnology fields. Currently, the most common preparation methods for hydrogels include chemical cross-linking [20], physical cross-linking [21], and radiation cross-linking [22]. Physical cross-linking was affected by temperature and had poor mechanical strength. The chemical cross-linking structure was durable but required an initiator or catalyst. Radiation cross-linking, with no initiator added, resulted in a product that was pure and dose safe. Therefore, in order to promote the application of hydrogels in various fields, different preparation methods were explored to improve the effective performance of the hydrogel preparations.

The quaternary ammonium salt of chitosan (HACC) is abundant in quaternary ammonium salt groups, which are strong cationic groups, thereby enhancing the positive charge of HACC. The cations present on the surface of HACC could electrostatically adsorb DNA molecules, making it an effective material for DNA adsorption. Additionally, HACC could establish electrostatic interactions with negatively charged components on bacterial surfaces, exhibiting potent antibacterial activity [23]. Polyvinyl alcohol (PVA) is a polymer widely utilized in multifunctional materials due to its commendable biocompatibility, hydrophilicity, flexibility, high transparency and degradability. Polyethylene glycol (PEG), possessing good biocompatibility and strong water solubility properties, helped maintain DNA stability while reducing non-specific adsorption and enhancing DNA adsorption efficiency. PEG has found extensive applications in gene delivery and drug delivery; furthermore, it had been demonstrated to “enhance the solubility and transfection properties of DNA” within the biomedical field [24,25]. A composite material is a new type of material that combines two or more different materials and has superior comprehensive properties. Composites could optimize specific adsorption properties by adjusting the composition and structure to meet various application requirements [26,27,28].

Electron beam radiation-induced preparation of hydrogels is a highly efficient ionic crosslinking technique that achieves polymer crosslinking of hydrogels through ionization and excitation effects. The operation is simple and the reaction speed is fast [29,30,31]. In this study, a biologically compatible, high adsorption capacity, structurally stable, and easily regenerable PVA/PEG/HACC composite material was prepared using electron beam radiation-induced hydrogel preparation. The prepared hydrogel was characterized by a series of tests to confirm the successful preparation of the PVA/PEG/HACC composite material. Then, the purification of ctDNA was achieved through adsorption experiments, and the possible adsorption mechanism was revealed. Furthermore, the optimal adsorption conditions were optimized, and the high adsorption of ctDNA was achieved, further confirming the application prospect of hydrogels in the field of adsorption. In addition, the antibacterial performance and cell viability of the hydrogel were evaluated by bacterial and cell experiments, further confirming the huge potential of hydrogels in the field of biomedicine.

## 2. Experimental Sections

### 2.1. Materials

HACC, PVA, and PEG were obtained from Shanghai Aladdin Biochemical Technology Co. (Shanghai, China). ctDNA was provided by Beijing Solaibao Technology Co. (Beijing, China). Peptone, yeast extract powder, beef extract and agar powder were from Sigma (St. Louis, MO, USA); *Escherichia coli* (*E.coli*) (CCTCCAB91112) and *Staphylococcus aureus* (*S. aureus*) (CCTCCAB910393) were from Wuhan University (Wuhan, China). High-sugar DMEM medium, double antibody, fetal bovine serum, PBS buffer solution, Hoechst 3342 staining solution, PI staining solution, 3-(4,5-dimethylthiazol-2-yl)-2,5-diphenyltetrazolium bromide (MTT), and ALP staining solution were from Wuhan Pnosay Life Science and Technology Co. (Wuhan, China). Isopropyl alcohol and ethanol were from National Pharmaceuticals (Beijing, China), and the high salt resolution solution was purchased from Kanglang Company (Shenzhen, China).

### 2.2. Radiation Construction of PVA/PEG/HACC Composite Hydrogel

**Figure 1:**
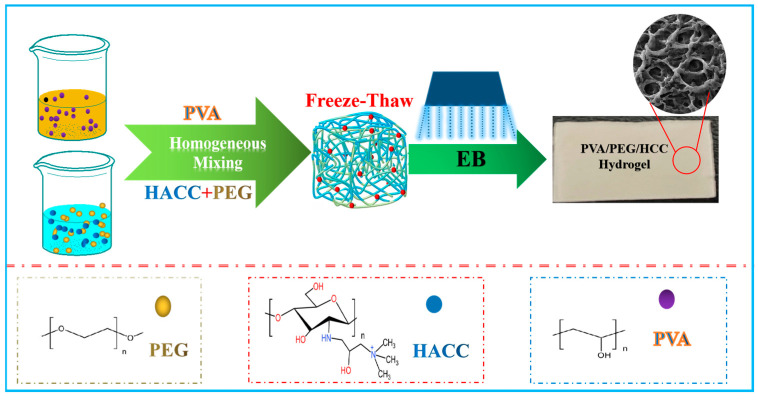
A schematic diagram of the preparation process of PVA/PEG/HACC hydrogel.

The preparation process of the PVA/PEG/HACC hydrogel is shown in Figure 1. The hydrogel was prepared with a concentration of 10% PVA, HACC (1–10)%, and 5% PEG, and radiated using a 1 MeV electron accelerator with a dose rate of 5 kGy/pass and a total dose of (5–25) kGy. The radiated samples were soaked in deionized water to remove unpolymerized material, and the PVA/PEG/HACC hydrogel samples were prepared. The radiation preparation mechanism used high-energy electron beam radiation to interact with HACC molecules, exciting their intramolecular carbon-nitrogen double bonds, opening the primary structure, and forming new free radicals. The cross-linking reaction was initiated by the reaction of free radicals with functional groups in PVA and PEG, resulting in the formation of a three-dimensional network structure among HACC, PVA, and PEG. 

### 2.3. Material Characterization

FTIR spectroscopy was used to analyze the chemical structure and functional groups of the composites; XRD was used to study the crystal structure and crystallographic properties of the materials; and TGA was used to measure the mass change of the materials during the heating process to reveal their thermal properties and stability. Scanning electron microscopy (SEM) was used to observe the surface morphology and microstructure of the materials. BET was used to analyze the specific surface area of the materials to demonstrate their adsorption properties and pore structure. The gel fraction test could be used to understand the radiation cross-linking of the material. The swelling ratio test was used to understand the water absorption and water retention properties of the hydrogel.

### 2.4. Study on the Adsorption Properties of Hydrogel to ctDNA

#### 2.4.1. Calf Thymus ctDNA Standard Curves Were Drawn

**Figure 2:**
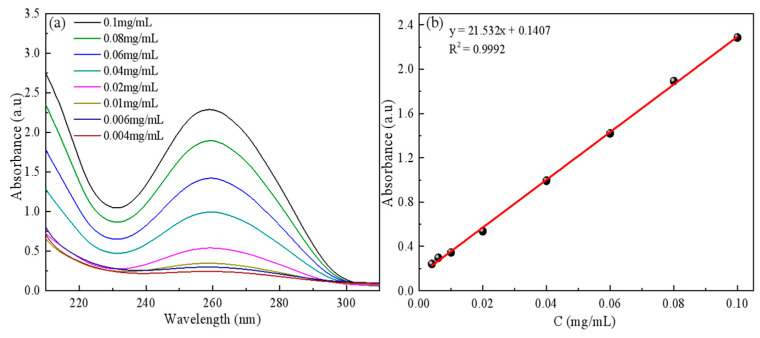
Standard curve of ctDNA: (**a**) UV absorption spectrum of ctDNA, (**b**) Fitting equation of ctDNA data.

The temperature of 293.15 K was maintained as 50 mg ctDNA was placed in a beaker, and an appropriate volume of PBS (pH = 7.4) was utilized as the solvent to prepare ctDNA standard solutions with concentrations ranging from 0.004 to 0.1 mg/mL. The maximum absorption wavelength of ctDNA at 260 nm was detected by UV spectrophotometry, and the standard curve of concentration versus absorbance was plotted (Figure 2).

#### 2.4.2. Adsorption Experiments of ctDNA

To demonstrate the adsorption capacity of PVA/PEG/HACC hydrogel for purification and isolation of ctDNA, 0.1 g of dry hydrogel was taken as adsorbent and added to the configured solution (0.25 mg/mL, 20 mL, pH = 7.45) in an iodine measuring flask. The adsorption experiment was conducted at 293.15 K for 180 min. The UV spectrophotometry of ctDNA was measured from the supernatant and the adsorption value was calculated.

Firstly, the comparison experiment of monadic, binary and ternary adsorption was carried out. Secondly, the radiation dose rate (5–30) kGy, the different proportion of HACC (1–10)%, and the initial concentration (0.125–6) mg/mL were adjusted to conduct adsorption experiments at 293.15 K, 298.15 K, and 310.15 K on the absorption performance of ctDNA by used gel samples. Then, the charge properties of the materials were determined by varying the initial concentration and adjusting the pH (5–12) using the zeta potential.

### 2.5. Biological Activity of PVA/PEG/HACC Ternary Composite Hydrogel

#### 2.5.1. Bacterial Culture

Preparation of liquid medium: Sodium chloride (2 g), peptone (4 g), and beef extract (1.2 g) were dissolved in 400 mL deionized water, the pH was adjusted to 7.0, and then it was autoclaved in an autoclave for 30 min. To prepare the bacterial buffer solution, NaCl (2 g), KCl (0.05 g), NaHPO_4_·12H_2_O (0.907 g), and KH_2_PO_4_ (0.06 g) were dissolved in 250 mL deionized water and autoclaved at 121 °C for 25 min in a high-pressure steam sterilizer. For *E. coli* and *S. aureus* cultures, ultraviolet sterilization was carried out on a super-clean table for 30 min, and 1 mL of bacterial solution stored at 4 °C was added to 25 mL of nutrient solution medium, and it was cultured at 37 °C and 180 rpm for 24 h.

#### 2.5.2. Preparation of the Gel Extract

Preparation of gel extract: PVA/PEG/HACC composite gels were prepared (96-well plates were used as molds to prepare gel trays) and sterilized by electron accelerator radiation. Then, 2 mL medium was added to each Petri dish and cultured in a cell incubator for 48 h to prepare the gel extract.

#### 2.5.3. Determination of Antibacterial Properties of Gels

The antibacterial properties of PVA/PEG/HACC composite hydrogels were tested by immersing them in deionized water and irradiating them with a UV lamp for 30 min. The gel sheets were then transferred to a Petri dish and their surface was evenly coated with 2 mL bacterial medium. They were placed in a constant temperature incubator at 37 °C, and the number of colonies was counted by an automatic colony counter at regular intervals to observe the growth and distribution of the colonies.

#### 2.5.4. Cell Toxicity Test, Cell Apoptosis Test 

MTT assays were used to measure cell viability, and Hoechst 33342 and PI staining were used to detect cell apoptosis. Mouse osteoblasts (MC3T3-E1) in the logarithmic growth phase were diluted to the target concentration and cultured in 96-well or 6-well plates. After the cells were attached, the culture medium was replaced. After washing with PBS, the blank sample and the control group had fresh culture medium added, and the experimental group had extracts of different ratios of gel added. The cytotoxicity was measured by a microplate reader, and the apoptosis was observed and photographed by an inverted fluorescence microscope. 

#### 2.5.5. Statistical Analysis 

Three parallel experiments were set up for each group of data, and the data are expressed as mean ± standard deviation. Statistical differences between the two groups were assessed using one-way ANOVA SPSS 17.0 software. *p* < 0.05 was considered to indicate statistical significance.

## 3. Results and Discussion

### 3.1. Radiation Preparation of Composite Hydrogel Proof

#### 3.1.1. FTIR Analysis

**Figure 3:**
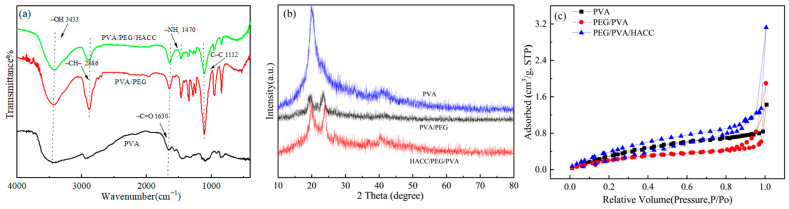
Characterization of different hydrogel samples: (**a**) FTIR spectra, (**b**) XRD patterns, (**c**) BET.

The infrared spectra of different hydrogel materials can be seen in Figure 3a. The stretching vibration of the hydroxyl group is at 3433 cm^−1^ in PVA [32], and the C=O carbon-oxygen bond vibration is at 1630 cm^−1^ [33]. The peak at 2886 cm^−1^ stems from the stretching vibration of C–H bonds in PEG, while the peak at 1112 cm^−1^ is the vibration of C–O–C bonds in PEG [34]. Finally, the peak at 1470 cm^−1^ indicates the symmetric tensile vibration peak caused by the quaternary ammonium salt group –NH_2_ [35]. The successful preparation of PVA/PEG/HACC hydrogel was demonstrated by FTIR spectroscopy.

#### 3.1.2. XRD Analysis

As shown in Figure 3b, the XRD spectrum of PVA shows a broad peak at around 2*θ* = 20° corresponding to the semi-crystalline nature of pure PVA as a (110) crystalline reflectance peak. The presence of this broad peak indicates the presence of a transition region between the crystalline and amorphous portions of the PVA sample, which shows strong scattering intensity [36]. PEG shows a broad peak around 2*θ* = 25°. This is due to reflections from the (110) crystal plane in PEG crystals, which are reflections of scattered and diffracted X-rays caused by the nuclei and electrons of the carbon element in the crystal [37]. HACC is an inorganic substance without fixed peak surfaces at 2*θ* values. Since HACC often exists in an amorphous state, its molecular structure does not exhibit a regular crystalline lattice arrangement. In amorphous polymers, due to the lack of a long-range ordered arrangement between molecules, X-rays are not reflected by regular structures and do not produce distinct crystal plane reflections, nor do they result in specific 2*θ* values [38]. Therefore, for polymers in which HACC is usually in an amorphous state, X-ray diffraction analysis often fails to observe significant crystalline reflections.

#### 3.1.3. Specific Surface Area Test (BET)

The BET data of different hydrogels are shown in Figure 3c. The BET adsorption curve reveals that PVA exhibits a type II isotherm, devoid of any discernible hysteresis loop. Conversely, both PVA/PEG and PVA/PEG/HACC exhibit a type IV isotherm with evident hysteresis loops, indicating the presence of highly irregular pore structures. The specific surface area and pore volume of the ternary composite hydrogels increased significantly, the pore size decreased, and the pores became denser. Because its PVA molecules formed a tight network structure through a cross-linking reaction, the structure restricted the degree of freedom of PVA molecules, resulting in a small hydrogel volume. The molecular weight of PEG was used to increase the expandability and tensile resistance of the binary material to regulate the pore size of the hydrogel and adjust the pore structure of the material. The presence of HACC, a cationic chitosan derivative, enhanced the cross-linking density and stability of the PVA/PEG/HACC hydrogel. As HACC acts as a positively charged chitosan derivative, it improved the cross-link density and stability of the hydrogel. The PVA/PEG/HACC hydrogel exhibited the highest specific surface area among all of the materials compared in Table 1, thereby enhancing its capacity for ctDNA adsorption. The positively charged quaternary ammonium salt groups in HACC interacted electrostatically with functional groups such as hydroxyl in the PVA/PEG and formed hydrogen bonds, which enhanced the cross-linking inside the hydrogel and increased the specific surface area, thereby increasing the amount of ctDNA adsorbed.

#### 3.1.4. Gel Fraction and Swelling Performance Test

**Figure 4:**
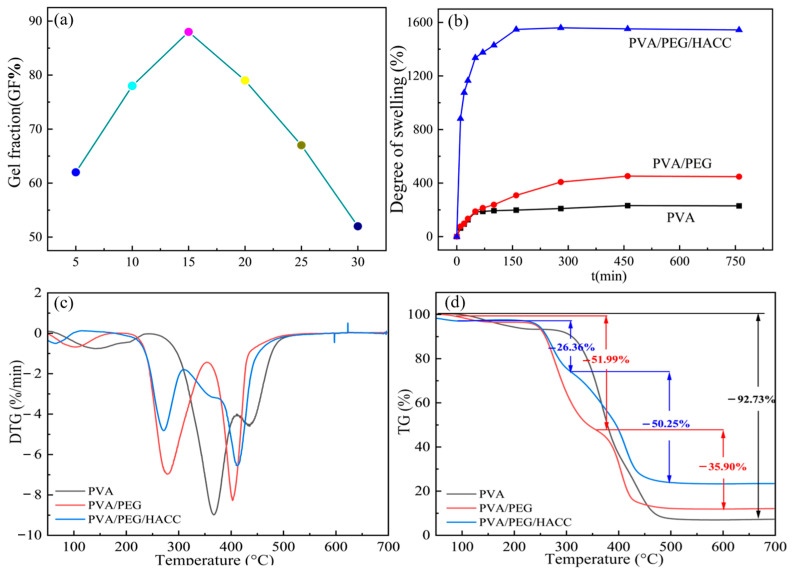
Testing of different hydrogel samples: (**a**) gel fractions of PVA/PEG/HACC, (**b**) swelling performance, (**c**) DTG curves, (**d**) TG curves.

As shown in Figure 4a, the best cross-linking effect of the PVA/PEG/HACC hydrogel was achieved when the radiation absorbed dose was at 15 kGy, and its gel fraction could reach 88%. Radiation-induced free radicals initiated cross-linking reactions in the hydrogels. PVA in PEG and HACC formed covalent bonds with the other functional groups to form a uniform and stable three-dimensional cross-linked network structure. This cross-linking not only enhanced the properties and solubility of the gel but also constructed a three-dimensional network structure through new chemical bonds. Ether bonds (–C–O–C–) or ester bonds (–COOC–) enhanced the stability and swelling properties of the gel. Due to free radicals, the gel molecules broke and degraded, the cross-linking density decreased, the chemical bond structure changed, and the gel stability and gel fraction decreased.

As shown in Figure 4b, the comparison results of the swelling ratios of different hydrogels showed that the PVA/PEG/HACC hydrogel had a higher swelling force, and its swelling rate could reach 1558%. The swelling rate of the hydrogel increased dramatically during the first 50 min, followed by a gradual stabilization to equilibrium over 3 h. The swelling rate of the hydrogels increased with the addition of PEG and HACC, and the pore structure of PEG promoted the water absorption and water retention of water molecules. Due to the hydrophilicity and ion exchange properties of HACC, the permeability and swelling ability of the water molecules were improved, and the swelling rate was further improved.

#### 3.1.5. Thermogravimetric Analysis (TGA)

The derivative thermogravimetry (DTG) curves of different samples in the temperature range of 50–700 °C are shown in Figure 4c. The mass loss of PVA samples occurred in the range of 350–400 °C, primarily due to the dehydration reaction of the molecular chains, resulting in changes in the polymer structure and partial degradation. This process was influenced by the hydrogen–oxygen bond inside the PVA molecule, which accelerated the structural destruction of the polymer chain. The binary materials were degraded twice at 250–300 °C and 400 °C, respectively; the former was mainly caused by a partial dehydration reaction of PVA and PEG molecular chains, and the latter was a more intense thermal cracking process. Rapid degradation of ternary materials in the range of 250–300 °C occurred. It was mainly affected by the partial dehydration reaction of PVA, PEG, and HACC molecular chains. At the rapid degradation stage at 400 °C, the degradation rate increased dramatically due to the more intense pyrolysis process, which led to the breaking of polymer chains and the release of gas products. Therefore, the degradation process at different temperature ranges was influenced by the dehydration reaction and thermal cleavage process, resulting in differences in the degradation rate.

Figure 4d shows the TG curves of different samples in the temperature range of 50–700 °C. In the first stage 50–300 °C, there was a 26.36% weight loss. The decline in quality was due to changes in the molecular structure of the hydrogel caused by the increase in temperature, including water evaporation and polymer chain breakage. In the second stage, 300–500 °C, there was a 50.25% weight loss, and major structural disruption and volatile release continued. The greater mass loss occurred due to more molecular chain breaks and shedding of side chains of the material. In the third stage, 500–700 °C, 35.90% weight loss was caused by the stability of the residue and the challenging degradation process. The majority of volatile substances had already evaporated in the first two stages, leaving relatively stable compounds.

#### 3.1.6. SEM Analysis

**Figure 5:**
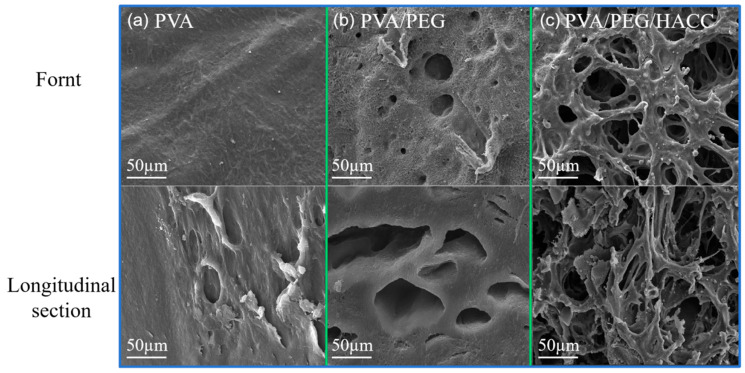
SEM images: (**a**) PVA, (**b**) PVA/PEG, (**c**) PVA/PEG/HACC.

The front and cross-section SEM images of different materials are shown in Figure 5a–c. The surface of the PVA hydrogel was uniform and smooth without obvious pore structures in the microstructure of the front and cross sections. Due to the chemical composition and crystal structure of PVA, it is not easy to form an obvious pore structure. So, the surface is flat and smooth. The front section of the binary hydrogel presents a certain pore structure. Due to the introduction of PEG, the pore structure of the hydrogel is more uniform. Because PEG enhanced the mobility of the inner chain of the hydrogel, functional groups such as hydroxyl groups underwent cross-linking reactions to construct a stable three-dimensional network structure. The front and cross section of the ternary hydrogel shows a good pore structure. Due to the positive charge of the quaternary ammonium group in HACC, the charge interaction between the functional groups in PVA and PEG materials increased the specific surface area and made the network structure more stable.

### 3.2. Adsorption Properties of HACC/PVA/PEG Hydrogel

#### 3.2.1. Data Analysis Methods

To investigate the effect of contact time on adsorption, adsorption experiments were performed to determine the equilibrium time of ctDNA adsorption on PVA/PEG/HACC hydrogels. The adsorption capacity Q_e_ (mg g^−1^) can be calculated as follows:

(1) $$Q_{e}=\frac{(C_{0}-C_{e})V}{m}$$

where Q_e_ (mg/g) represents the adsorption value at adsorption equilibrium, C_0_ (mg/mL) represents the concentration of ctDNA before adsorption, C_e_ (mg/mL) represents the concentration of ctDNA after adsorption, V (mL) represents the volume of the adsorbed solution, and m (g) denotes the mass of the adsorbent.

Adsorption kinetics is the study of the process of adsorption of substances on solid surfaces or interfaces. In adsorption, kinetics research focuses on the analysis of adsorption rates and mechanisms, mainly examining the change of adsorption with time and the kinetic mechanism behind it. To gain a deeper understanding of this process, different kinetic models were used for analysis, including the pseudo-first-order kinetic model and pseudo-second-order kinetic model, as well as the intraparticle diffusion model. The pseudo-first-order kinetic model equation is [39] as follows:

(2) $$In(Q_{e}-Q_{t})=InQ_{e}-k_{1}t$$

where Q_e_ (mg/g) represents the adsorption value at adsorption equilibrium, Q_t_ (mg/g) represents the adsorption value at the contact time, t represents the adsorption time, and *k*_1_ min^−1^ denotes the velocity constant. The equation of the pseudo-second-order kinetic model [40] is as follows:

(3) $$\frac{t}{Q_{t}}=\frac{1}{k_{2}.Q_{e}^{2}}+\frac{t}{Q_{e}}$$

where Q_e_ (mg/g) represents the adsorption value at adsorption equilibrium, Q_t_ (mg/g) represents the adsorption value at contact time, t represents the adsorption time, and *k*_2_ (g/(mg·min)) denotes the rate constant. The internal diffusion model [41] equation is expressed as follows:

(4) $$Q_{t}=k_{\mathrm{id}}.t^{1/2}+C$$

where Q_t_ denotes the adsorption value at a contact time, t denotes the adsorption time, and *k_id_* (g·min^1/2^) denotes the velocity constant. The adsorption isothermal model is a mathematical model to study the adsorption process in which the adsorption and desorption between adsorbent and solid surface reach equilibrium. Common adsorption isothermal models include the Langmuir model and the Freundlich model. The study of this model aims to analyze the relationship between adsorbent and adsorbate in adsorption systems in terms of adsorption amount under different conditions, exploring the physical and chemical interactions between adsorbed molecules and solid surfaces. The expression is as follows:

(5) $$\frac{C_{e}}{Q_{e}}=\frac{C_{e}}{Q_{m}}+\frac{1}{K_{L}.Q_{m}}$$

where C_e_ (mg/mL) represents the adsorption value at adsorption equilibrium, Q_e_ (mg/g) represents the adsorption value at adsorption equilibrium, Q_m_ (mg/g) represents the maximum adsorption capacity, and *K_L_* (mL/mg) denotes the model constant. Through the adsorption process [42], R_L_ parameters can evaluate the Langmuir model, and the formula is as follows:

(6) $$R_{L}=\frac{1}{1+K_{L}\times C_{0}}$$

where the R_L_ value represents the ease of adsorption, with the process categorized as irreversible. (R_L_ = 0) represents irreversible adsorption, adsorption that quickly occurred (0 < R_L_ < 1), and adsorption that did not promptly occur (R_L_ > 1). The Freundlich model is one of the empirical models for describing adsorption processes on non-homogeneous surfaces. The expression is as follows:

(7) $$InQ_{e}=InK_{F}+\frac{1}{n_{F}}InC_{e}$$

where Q_e_ (mg/g) represents the adsorption value at adsorption equilibrium, C_e_ (mg/mL) represents the concentration after adsorption, *K_F_* (mL/mg) represents the model constant, 1/n_F_ represents the dimensionless constant of the adsorption strength, and the 1/n_F_ value indicates the difficulty of the adsorption process.

To further investigate the adsorption mechanism, we studied the adsorption thermodynamics of hydrogel adsorbents. The study of adsorption thermodynamics allows the adsorption process of adsorbent materials to be analyzed from an energetic point of view. The thermodynamic characteristics can be expressed by three parameters: Gibbs free energy (ΔG), enthalpy change (ΔH), and entropy change (ΔS) [43], as follows:

(8) $$InK=-\frac{\Delta H}{R}\times \frac{1}{T}+\frac{\Delta S}{R}$$

(9) $$K=\frac{Q_{e}}{C_{e}}$$

(10) ΔG=ΔH−TΔS

where K is the thermodynamic partition coefficient; Q_e_ is the amount adsorbed at equilibrium; C_e_ is the concentration of the adsorbent at equilibrium; R is the ideal gas constant, 8.314 J·moL^−1^·K^−1^; and T is the Kelvin temperature K. To check the desorption effect of ctDNA, desorption tests were performed on the adsorbed hydrogels, calculated as follows:

(11) $$D\%=\frac{C_{d}}{C_{0}}\times 100$$

where C_d_ indicates the ctDNA concentration (mL/mg) in the eluate and C_0_ indicates the original concentration of the adsorbed ctDNA (mL/mg).

#### 3.2.2. Effect of Different Samples on the Adsorption Efficiency of ctDNA

**Figure 6:**
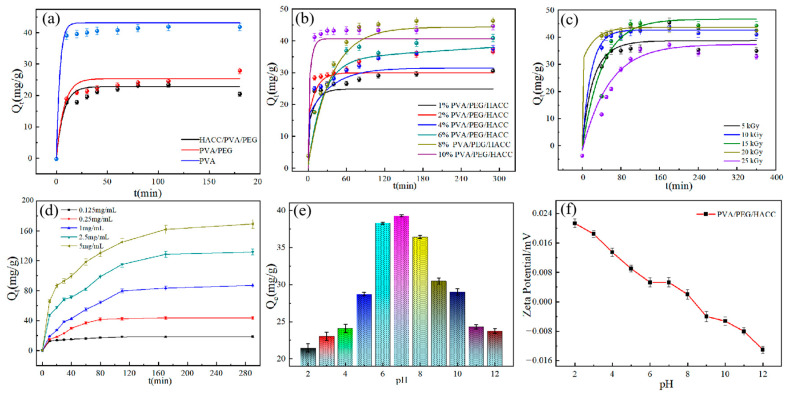
Influence of different factors on ctDNA absorption of PVA/PEG/HACC hydrogel: (**a**) different samples; (**b**) different HACC content; (**c**) different radiation absorption doses; (**d**) different initial concentrations; (**e**) different pH values; (**f**) zeta potentials at different pH values.

As shown in Figure 6a, the ternary composite hydrogels had the best attachment effect on ctDNA, as concluded from the adsorption comparison experiments. The PVA hydrogel and binary hydrogel mainly interacted with ctDNA through physical interactions. However, the small pore size structure of the material, the lack of active sites on the surface, and the absence of active functional groups led to poor affinity with ctDNA, reducing the adsorption effect. The ternary hydrogel, on the other hand, adsorbed ctDNA through both electrostatic and physical effects since the positively charged quaternary ammonium salt functional group of HACC formed an electrostatic interaction with the phosphate group in ctDNA. Meanwhile, the hydroxyl groups contained within HACC not only strengthen the architecture of the hydrogels, but also significantly enhance the adsorption efficiency of the hydrogels towards ctDNA by forming hydrogen bonds with ctDNA. In addition, the addition of PEG changed the hydrogel structure and increased the pore structure and surface active sites, which further enhanced the adsorption of ctDNA by the hydrogel.

The effect of HACC monomer content on the adsorption of PVA/PEG/HACC hydrogel can be seen in Figure 6b. The best adsorption amount of 44.17 mg/g was achieved when the HACC content was 8%. Adsorption decreased when the quaternary ammonium salt concentration was below a certain threshold (below 8%). During adsorption, the ctDNA presented a negative charge, while the quaternary ammonium salt was positively charged. When the concentration of quaternary ammonium salt was too low, it could not provide enough positive charge to interact with the negative charge of ctDNA, resulting in decreased adsorption. On the contrary, when the ammonium salt concentration exceeded a certain threshold, the ionic strength of the solution increased. The excessive positive charge of quaternary ammonium formed an unfavorable ionic complex, which resulted in a weakening of the adsorption capacity.

#### 3.2.3. The Effect of Different Radiation Doses on the Adsorption of ctDNA

As shown in Figure 6c, when the absorbed dose was 15 kGy, the adsorption effect was the best, and the adsorption capacity reached 45.97 mg/g. Due to the influence of different radiation absorption doses on the structural cross-linking degree of PVA/PEG/HACC hydrogel, the adsorption effect of ctDNA was affected. When the radiation dose was small, the degree of freedom of the internal chain of the hydrogel increased, resulting in insufficient cross-linking and affecting the adsorption performance. When the absorbed radiation dose was 15 kGy, better cross-linking could be induced. An appropriate radiation absorption dose activated the cross-linking reaction of the polymer molecules and promoted the cross-linking reaction within the polymer molecules. However, the radiation absorbed dose at 20 kGy exceeded the cross-linking threshold of the material. The cross-linking structure became loose, and even the fracture of the cross-linking chain affected the cross-linking effect, resulting in reduced adsorption of ctDNA. However, the radiation absorbed dose at 20 kGy exceeded the cross-linking threshold of the material. The cross-linking structure became loose, and even the fracture of the cross-linking chain affected the cross-linking effect, resulting in reduced adsorption of ctDNA.

The effect of PVA/PEG/HACC hydrogel on ctDNA adsorption at a specific temperature was examined and is shown in Figure 6d. The initial concentration ranged from 0.125 to 5 mg/mL, and the adsorption capacity exhibited a positive correlation with the increase in initial concentration. When the concentration was 2.5 mg/mL, the adsorption capacity reached 131.77 mg/g, and the adsorption capacity remained stable. This was mainly due to the low affinity between adsorption interactions at low initial concentrations, resulting in a low adsorption capacity. During the initial stage of adsorption, as the initial concentration increased, the ctDNA molecules in the solution bound to more adsorption sites in the hydrogel material, and the adsorption rate also increased rapidly. As the adsorption process proceeds, the adsorption sites become saturated. When the number of molecules on the adsorption sites reaches a specific number, the adsorption rate begins to slow. Eventually, the adsorption process reaches a dynamic equilibrium; i.e., adsorption saturation.

#### 3.2.4. Effect of pH on Adsorption and Zeta Potential Determination

The pH value could regulate the charge degree, active sites, and structural characteristics of the adsorbate during the adsorption process. As shown in Figure 6e, the effect of pH on the adsorbent was investigated. The adsorption capacity of ctDNA in the range of pH 2–12 is known. The adsorption amount is 39.35 mg/g at pH 7–8. The adsorption decreases gradually at a pH greater than 8. Firstly, under neutral to weakly alkaline conditions, the quaternary ammonium salt group of PVA/PEG/HACC hydrogel maintained the best charge performance. It was more conducive to the electrostatic adsorption of the phosphate group of ctDNA. Secondly, the hydration properties of the solution under neutral to alkaline conditions made the interaction between quaternary chitosan and ctDNA more stable and favored the adsorption process. Thus, the adsorption of DNA by quaternary chitosan was maximized in the neutral to alkaline pH range [44].

To investigate the charge properties on the surface of the PVA/PEG/HACC hydrogel, the material was tested for zeta potential as shown in Figure 6f. At pH = 9, a zeta potential of −0.0056 mV was measured. The potential decreased with the increase of pH value, indicating that the hydrogel carried a large number of positively charged groups. These positive charges are more likely to attract the negative charges of ctDNA, which is more conducive to effective adsorption.

#### 3.2.5. Adsorption Kinetics Studies

**Figure 7:**
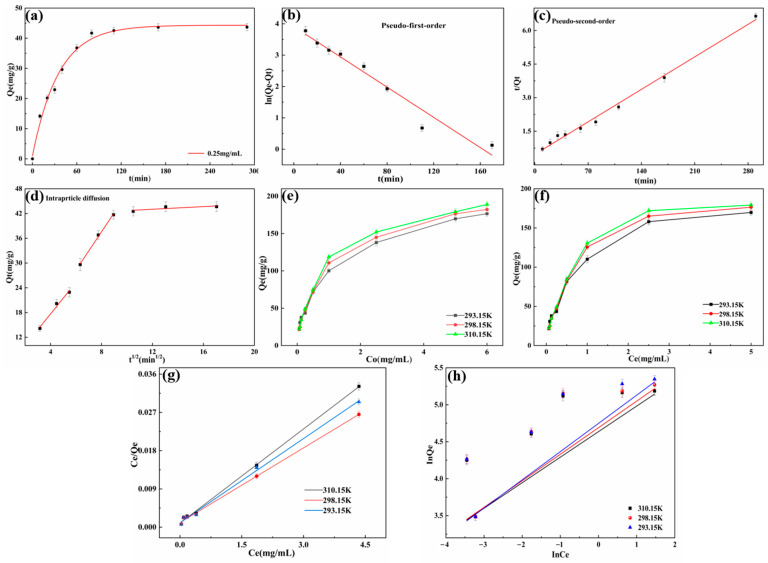
(**a**) Influence of contact time on ctDNA absorption by PVA/PEG/HACC hydrogel (pH = 7.35, T = 293.15 K), The kinetics model of ctDNA absorption on PVA/PEG/HACC hydrogel: (**b**) Pseudo-first-order kinetics; (**c**) Pseudo-second-order kinetics; (**d**) Particle diffusion model fitting; (**e**) Influence of the initial concentration and temperature on ctDNA absorption by PVA/PEG/HACC hydrogel; (**f**) Isotherms of ctDNA adsorption by PVA/PEG/HACC hydrogel; (**g**) Langmuir isotherm model fitting by PVA/PEG/HACC hydrogel; (**h**) Freundlich isotherm model fitting by PVA/PEG/HACC hydrogel.

Adsorption time is an essential factor that plays a crucial role in adsorption performance. The trend of adsorption at different times is shown in Figure 7a. The PVA/PEG/HACC water gel adsorbed a quantity of ctDNA that quickly reached a balance within 80 min, with an adsorption capacity of 41.72 mg/g, because the water gel surface had active sites in the initial stage and was more unsaturated. The electrostatic, hydrogen bonds and van der Waals forces of interaction promoted the fast adsorption of ctDNA and then drove the rapid increase of the adsorption rate. The adsorption amount slowly increased to 43.63 mg/g from 110 min to 170 min, and finally, the adsorption amount of 48.67 mg/g remained stable for another 290 min. In the slow stage of adsorption, the active sites of the hydrogel were gradually occupied by ctDNA molecules, and the adsorption reached a saturated state. The adsorption rate gradually slowed down and eventually stabilized.

In order to further explore the possible mechanism of ctDNA adsorption on PVA/PEG/HACC hydrogels, the experimental data were analyzed using the adsorption kinetic model (Table 2). As shown in Figure 7b,c, after fitting the pseudo-primary and pseudo-secondary kinetic models, the pseudo-secondary kinetic model showed a good fit in the adsorption process, with a model fit of 99.38%. In addition, the adsorption capacity predicted by the pseudo-secondary kinetic model was quite close to the measured results, further confirming that the adsorption process was chemisorption. As shown in Figure 7d, the results of the adsorption data fitting of ctDNA on the PVA/PEG/HACC hydrogel indicated three phases of adsorption. The first one was rapid adsorption. In this stage, there were mainly many vacant adsorption sites on the surface of the adsorbent, and the ctDNA could be quickly adsorbed to the adsorbent through the adsorption sites. The second stage was the process of gradual adsorption, because its adsorption sites on the surface of the adsorbent had been occupied by the first arriving ctDNA. Therefore, the remaining ctDNA can only rely on diffusion to complete adsorption within the materia. The adsorption process in this stage was a process of diffusion from the surface of the adsorbent to the interior, and therefore, the rate of adsorption decreased compared to the first stage. The third stage was the adsorption equilibrium stage, where both the surface and the interior of the adsorbent were occupied by ctDNA, and the adsorption reached equilibrium. The adsorption internal diffusion data were fitted, and the results are shown in Table 3. It was proved that there was no single intra-granular diffusion among the three stages, and other factors affected the adsorption rate [45].

#### 3.2.6. Adsorption Isotherm Studies

To study the relationship between the adsorption capacity of the adsorbent and the adsorbate in the equilibrium state and the concentration in the solution, the effect of initial ctDNA concentration on adsorption at different temperatures was measured, as shown in Figure 7e,h. At a specific temperature, the adsorption capacity changed from a rapid increase to a slow rise with the growth of the initial concentration and finally reached the adsorption equilibrium. The higher the temperature, the more pronounced this trend was, as higher initial concentrations of ctDNA drove the adsorbent to adsorb more quickly, resulting in a higher affinity between the adsorbent and the adsorbate. The adsorption of the adsorbent did not change significantly at 293.15 K and 298.15 K, but it increased substantially up to 169.37 mg/g at 310.15 K. The adsorption of the adsorbent at 293.15 K and 298.15 K was insignificant. At the same time, the linear fit correlation coefficients of the Langmuir equation were 0.9968, 0.9981, and 0.9984. The fitted data of the Langmuir model showed a higher linear correlation and better fit. This suggested that the adsorption of ctDNA on the PVA/PEG/HACC hydrogel was dominated by a monolayer adsorption process. At 310.15 K, fitting was used to obtain the maximum adsorption capacity of 169.37 mg/g. It was close to the experimental result of 163.93 mg/g, indicating that the adsorption of ctDNA by PVA/PEG/HACC hydrogel conformed to the Langmuir model. The K_L_ values at different temperatures were between 0 and 1, indicating that the adsorption process of ctDNA on PVA/PEG/HACC hydrogel was easy to carry out [46].

#### 3.2.7. Adsorption Thermodynamic Studies

Adsorption thermodynamic analysis revealed the thermodynamic nature of the adsorption process and analyzed the effect of various factors on the adsorption process. Based on the fitting data in Table 4, the following conclusions were drawn. Negative values of ΔG indicated that adsorption occurred spontaneously. ΔG decreased with increasing temperature, suggesting an increase in temperature was more favorable to the adsorption process. In addition, the positive ΔH showed that the adsorption process was an endothermic reaction. The adsorption rate of ctDNA on the PVA/PEG/HACC hydrogel exhibited an increase with rising temperature within the range of 293.15–310.15 K, which could be attributed to the augmentation of active sites as the temperature increased, while ΔS was positive, indicating that adsorption was a process of increasing entropy and increasing degree of disorder. At the same time, the adsorption of ctDNA increased the degree of freedom of the whole system.

#### 3.2.8. ctDNA Desorption and Hydrogel Regeneration Performance Tests

**Figure 8:**
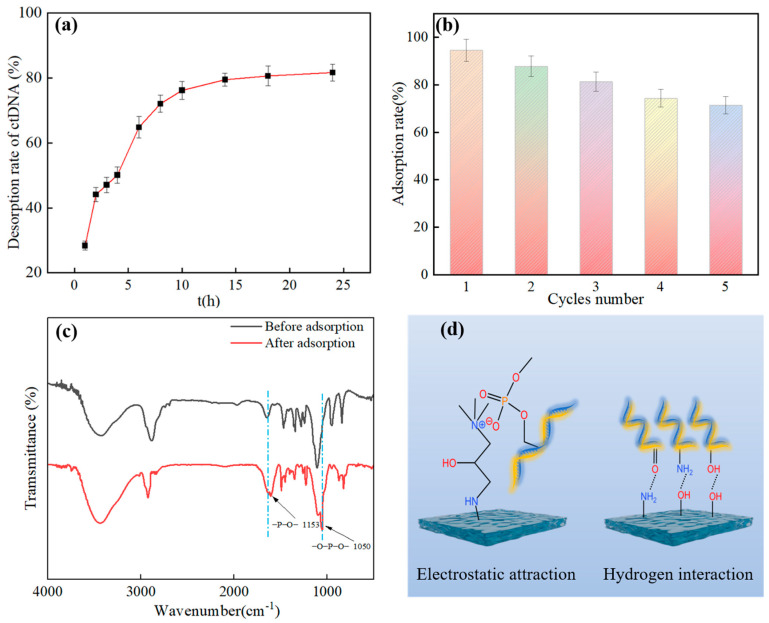
PVA/PEG/HACC hydrogel: (**a**) ctDNA desorption; (**b**) hydrogel recycling test; (**c**) FTIR spectra before and after ctDNA adsorption by PVA/PEG/HACC hydrogel; (**d**) Possible mechanism of ctDNA adsorption by PVA/PEG/HACC hydrogel.

As shown in Figure 8a, the adsorbed hydrogel was desorbed in the resolving solution for 24 h at room temperature with stirring. A high salt resolution solution was selected for the elution process of ctDNA from the PVA/PEG/HACC hydrogel. The adsorbent was washed twice using 1 mL each of 80% isopropanol and 70% ethanol, with a final elution rate of 81.67%. The salt ions in the high salt resolution solution could shield the electrostatic interaction between ctDNA and the surface, weaken the adsorption force, and promote the effective elution of ctDNA. In addition, the washing conditions of 80% isopropanol and 70% ethanol affected the binding force between the hydrogel and the ctDNA, contributing to the washing efficiency. To explore the cyclic renewability of the adsorbent, as shown in Figure 8b, the adsorption capacity was 71.39% after undergoing five adsorption–desorption cycles. The main reasons for this were the presence of depletion of the adsorbent from multiple biological cycles, a decrease in the number of active sites, and a slight change in the pore size of the hydrogel. The adsorption–desorption experiments showed that the PVA/PEG/HACC hydrogel had good cyclic regeneration properties.

#### 3.2.9. Adsorption Mechanism Analysis

Figure 8c shows the FTIR spectra of the PVA/PEG/HACC hydrogels before and after ctDNA adsorption. The adsorbed hydrogel peaked at 1153 cm^−1^ for ctDNA phosphodiester bonds, generated by vibrations induced by deoxyribose and phosphate groups inside the phosphodiester bonds. At the same time, the ctDNA phosphate group at 1050 cm^−1^ also appeared. Within the family, it was mainly composed of phosphorus in vibrations and phosphorus–oxygen–p-oxygen peaks. The above results confirmed that ctDNA was adsorbed by PVA/PEG/HACC hydrogel [47].

As shown in Figure 8d, its possible mechanism of adsorption was mainly through electrostatic interactions, hydrogen bonding, and van der Waals forces. The electrostatic interaction was primarily through the formation of electrostatic interactions between the quaternary ammonium ions in quaternary chitosan and the phosphate groups of ctDNA [48]. As shown in Figure 6f, the zeta potential proved that the PVA/PEG/HACC composite hydrogel was positively charged, which enabled it to better form electrostatic interactions with the phosphate group of ctDNA. The adsorption mechanism worked through van der Waals forces and hydrogen bonding between hydroxyl and oxygenated groups in PVA and PEG and ctDNA bases [49]. Finally, as shown in Figure 5a–c, the scanning electron microscope showed the rich pore structure of the PVA/PEG/HACC composite hydrogel, which provided conditions for the adsorption of ctDNA through the pores of the hydrogel.

### 3.3. Study of the Bioactivity of PVA/PEG/HACC Hydrogel

#### 3.3.1. Hydrogel Antibacterial Experiment

**Figure 9:**
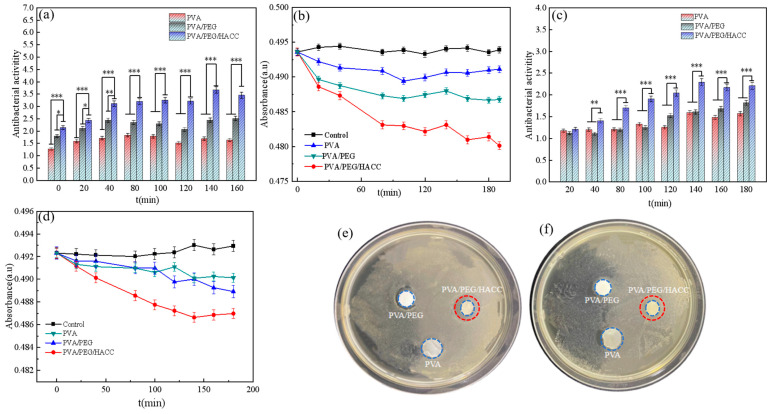
Antibacterial test. (**a**,**b**) Antimicrobial effect of *E. coli*; (**c**,**d**) Antimicrobial effect of *S. aureus*; (**e**,**f**) Bacteriostatic rings of different hydrogel samples against (**e**) *E. coli* and (**f**) *S. aureus*. The blue circles mark the location of the drugs, and the red marks the inhibition circles. Note: *: *p* < 0.05, **: *p* < 0.01, ***: *p* < 0.001.

As shown in Figure 9a,b, the ternary composite hydrogel has a specific antibacterial effect on *E. coli* with an antibacterial rate of 41.70%. The negatively charged cell membrane of *E. coli* can interact with the positively charged quaternary ammonium salt, thereby influencing the structure and function of the bacteria. At the same time, quaternary ammonium salt interfered with essential pathways such as nucleic acid, lipid, and glucose metabolism by affecting the active ingredients in the metabolic pathway of *E. coli*, thereby affecting the transcription process of protein synthesis, thereby affecting the growth and viability of *E. coli* to achieve an antibacterial effect [50].

As shown in Figure 9c,d, the ternary composite hydrogel also showed a certain antibacterial activity against *S. aureus*, with an antibacterial activity of 43.41%. This was because the complex cell wall structure of *S. aureus* contained multiple layers of dextran and peptidoglycan. Components such as quaternary ammonium salts in the hydrogel interfered with cell wall synthesis in this bacterium, leading to cell wall disruption and compromising bacterial integrity and function. In addition, *S. aureus* often forms biofilms, and hydrogels reduced its viability and infectivity by disrupting the biofilm structure or inhibiting its growth. Therefore, the quaternary ammonium salt component of the hydrogel disrupted the cell wall synthesis of *S. aureus* and affected its survival and reproduction in the external environment. As shown in Figure 9e,f, the inhibitory circles indicate that the ternary composite hydrogel had obvious bacteriostatic effects on *E. coli* and *S. aureus*. Because of its quaternary ammonium structure, it could disrupt cell membranes and cause cell death. Also, it could make the cells produce oxygen free radicals, and the antiseptic effect of oxygen free radicals helped eliminate bacteria.

#### 3.3.2. Cell Compatibility

**Figure 10:**
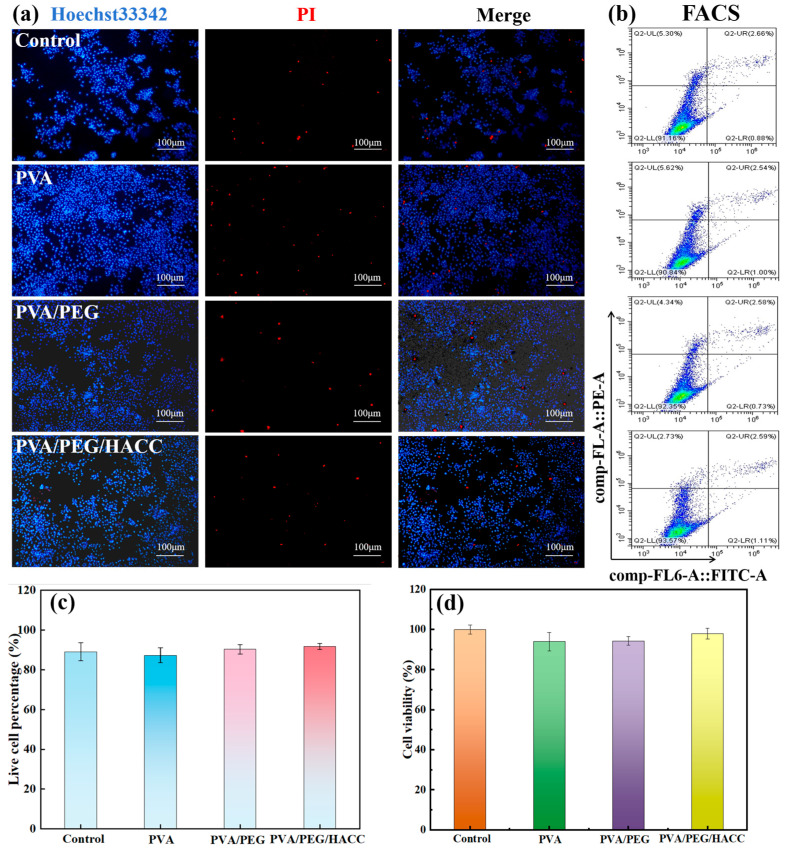
(**a**) Detection of MC3T3-E1 cell apoptosis by Hoechst33342/PI staining; (**b**) Flow cytogram; (**c**) Percentage of live cells in flow cytogram; (**d**) MTT assay for MC3T3-E1 cell activity in different samples.

PEG exhibited a stable molecular structure, good water solubility, non-irritation and low toxicity to biological tissues, as well as adjustable hydrotropism and hydrophobicity [51]. The quaternary ammonium salt group in HACC promoted cell adsorption and enhanced cell compatibility [52]. To assess the cytocompatibility of PVA/PEG/HACC hydrogel, Hoechst 33342/PI staining was employed to observe the ratio of live to dead MC3T3-E1 cells. Hoechst 33342 bound to nuclear DNA, producing blue fluorescence, while PI bound to cellular DNA, producing red fluorescence. As shown in Figure 10a, results from Hoechst33342/PI staining revealed less red fluorescence in the PVA/PEG/HACC group compared with the other groups, indicating a lower death rate for MC3T3-E1 cells, which also confirmed good cytocompatibility for PVA/PEG/HACC. Flow cytometry was used to detect apoptosis and assess cell function for a more comprehensive understanding of cell identity, function and state. As illustrated in Figure 10b,c, the apoptosis rates were all below 10% for the control, PVA, PVA/PEG and PVA/PEG/HACC groups with the lowest rate recorded at 6.43% for the latter group. The MTT cytotoxicity test was employed to assess the cytotoxicity, with the absorbance ratio between the experimental and control groups serving as an indicator of cell viability. These findings revealed that (Figure 10d) cell viability exceeded 90% in all groups, while reaching a remarkable 98% in the PVA/PEG/HACC group, primarily attributed to HACC’s ability to enhance cell adhesion and proliferation. Moreover, it facilitated an optimal microenvironment for cellular survival and functionality.

## 4. Conclusions

In summary, this study successfully developed a PVA/PEG/HACC hydrogel using an electron beam radiation method for ctDNA adsorption. The structure and morphology of the hydrogel were thoroughly characterized using FTIR spectroscopy, XRD, SEM, BET analysis, and TGA. The gel fraction was 88%, and the swelling rate reached a maximum at an absorbed radiation dose of 15 kGy. The BET surface area was 1.9510 m^2^/g. Adsorption tests demonstrated that the PVA/PEG/HACC hydrogel exhibited an adsorption rate of 89% for ctDNA under conditions of an initial ctDNA concentration of 0.25 mg/mL, a radiation dose rate of 15 kGy, a HACC monomer concentration of 8%, and a pH of 7–8. The adsorption data were analyzed and fitted using adsorption kinetic, isothermal adsorption, and adsorption thermodynamic models. The adsorption process followed the Langmuir isotherm and a pseudo-second-order kinetic model. At 310.15 K, the maximum ctDNA adsorption capacity of the hydrogel was 169.37 mg/g. Furthermore, the process was primarily characterized by monolayer chemisorption and influenced by intraparticle diffusion. The adsorption process was spontaneous, and high temperatures enhanced the adsorption efficiency. The maximum desorption rate of ctDNA was 81.67%. After five adsorption–desorption cycles, the adsorption rate remained at 71.39%. FTIR and Zeta potential analyses before and after adsorption indicated that the adsorption mechanisms involved electrostatic interactions, hydrogen bonding, and van der Waals forces. The hydrogel exhibited antibacterial properties, with antibacterial rates of 41.70% against *E. coli* and 43.41% against *S. aureus.* Cytotoxicity evaluations using MC3T3-E1, including MTT assays, apoptosis tests, and flow cytometry, indicated a cell viability of ~95% and an apoptosis rate of ~2.3%, confirming the biocompatibility of the hydrogel. These results establish a solid foundation for the use of PVA/PEG/HACC in DNA adsorption and various biomedical applications.

## Figures and Tables

**Table 1:** BET of different hydrogel samples.

Samples	Surface Area (m^2^/g)	Pore Volume (mL/g)	Pore Size (nm)	Adsorption Average Pore (nm)
PVA	1.0222	0.000952	117.233	7.5252
PVA/PEG	1.7001	0.001947	68.499	4.3378
PVA/PEG/HACC	1.9510	0.012090	48.622	4.6639

**Table 2:** Kinetic parameters of ctDNA adsorption on PVA/PEG/HACC hydrogel.

Kinetic Models	Parameters	Values
Pseudo-first order	K_1_	0.0349
	Qe	47.8609
	R^2^	0.976
Pseudo-second order	K_2_	9.1 × 10^4^
	Qe	48.3092
	R^2^	0.9938
Intra-particle diffusion	K_id1_	4.625
	C_1_	0.564
	R^2^	0.9930
	K_id2_	3.834
	C_2_	2.334
	R^2^	0.9633
	K_id3_	0.159
	C_3_	43.131
	R^2^	0.3228

**Table 3:** Isotherm parameters of ctDNA adsorption on PVA/PEG/HACC hydrogel.

Kinetic models	Parameters	T (k)		
		293.15	298.15	310.15
Langmuir	*Q*_m_ (mg/g)	135.135	149.254	163.934
	*K*_L_ (L/mg)	0.0555	0.0558	0.074
	*R* ^2^	0.9968	0.9981	0.9984
Freundlich	*K*_F_ (mg^1−1/n^·L^1/n.^g^−1^)	109.793	103.203	117.296
	*n*	2.606	2.795	2.894
	*R* ^2^	0.7771	0.7349	0.7285

**Table 4:** Isotherm parameters of ctDNA adsorption on PVA/PEG/HACC hydrogel.

Temperature (K)	ΔG (KJ/mol)	ΔH (KJ/mol)	ΔS (J/mol·K)
293.15	−16.564	68.238	289.280
298.15	−18.011		
310.15	−21.482		

## Data Availability

The data presented in this study are available on request from the corresponding author.

## References

[1] Dao J., Conway P.J., Subramani B., Meyyappan D., Russell S., Mahadevan D. (2023). Using cfDNA and ctDNA as oncologic markers: A path to clinical validation. Int. J. Mol. Sci..

[2] Eturi A., Bhasin A., Zarrabi K.K., Tester W.J. (2024). Predictive and prognostic biomarkers and tumor antigens for targeted therapy in urothelial carcinoma. Molecules.

[3] Temperley H.C., Fannon T., O’Sullivan N.J., O’Neill M., Mac Curtain B.M., Gilham C., Kelly M.E. (2024). Assessing circulating tumour DNA (ctDNA) as a biomarker for anal cancer management: A systematic review. Int. J. Mol. Sci..

[4] Shereef H.A., Moemen Y.S., Elshami F.I., El-Nahas A.M., Shaban S.Y., Eldik R. (2023). DNA binding and cleavage, stopped-flow kinetic, mechanistic, and molecular docking studies of cationic ruthenium (II) nitrosyl complexes containing “NS_4_” core. Molecules.

[5] Murray J.C., Sivapalan L., Hummelink K., Balan A., White J.R., Niknafs N., Anagnostou V. (2024). Elucidating the heterogeneity of immunotherapy response and immune-related toxicities by longitudinal ctDNA and immune cell compartment tracking in lung cancer. Clin. Cancer Res..

[6] Abdelrahim M., Esmail A., Saharia A., McMillan R., He A.R., Starr J.S., Ghobrial R.M. (2022). Feasibility of disease recurrence monitoring in liver post-transplantation for patients with hepatocellular carcinoma via personalized and tumor-informed ctDNA test. J. Clin. Oncol..

[7] Han X., Tang X., Zhu H., Zhu D., Zhang X., Meng X., Wang Z. (2022). Short-term dynamics of circulating tumor DNA predicting efficacy of sintilimab plus docetaxel in second-line treatment of advanced NSCLC: Biomarker analysis from a single-arm, phase 2 trial. Immunother. Cancer.

[8] Nabet B.Y., Esfahani M.S., Moding E.J., Hamilton E.G. (2020). Noninvasive early identification of therapeutic benefit from immune checkpoint inhibition. Cell.

[9] Cinar M., Martinez-Medina L., Puvvula P.K., Arakelyan A., Vardarajan B.N., Anthony N., Bernal-Mizrachi L. (2022). Retrotransposons facilitate the tissue-specific horizontal transfer of circulating tumor DNA between human cells. bioRxiv.

[10] Tsai C.J., Yang J.T., Guttmann D.M., Shaverdian N., Eng J., Yeh R., Powell S.N. (2022). Final analysis of consolidative use of radiotherapy to block (CURB) oligoprogression trial-a randomized study of stereotactic body radiotherapy for oligoprogressive metastatic lung and breast cancers. Int. J. Radiat. Oncol. Biol. Phys..

[11] Arisi M.F., Dotan E., Fernandez S.V. (2022). Circulating tumor DNA in precision oncology and its applications in colorectal cancer. Int. J. Mol. Sci..

[12] Yao W., Mei C., Nan X., Hui L. (2016). Evaluation and comparison of in vitro degradation kinetics of DNA in serum, urine and saliva: A qualitative study. Gene.

[13] Janabi A.H., Kerkhof L.J., McGuinness L.R., Biddle A.S., McKeever K.H. (2016). Comparison of a modified phenol/chloroform and commercial-kit methods for extracting DNA from horse fecal material. J. Microbiol. Methods.

[14] Shi J.J., Wu D., Liu T.Z., Hao S.J., Meng B.C., Li S.L., Liu Y.N. (2022). Comparative of forensic DNA iIdentification using Cell lysis method and magnetic beads method. Fa Yi Xue Za Zhi.

[15] Chakka R., Vadaguru Dakshinamurthy R., Rawal P., Belladamadagu Appajappa S., Pramanik S. (2023). Gallic acid a flavonoid isolated from euphorbia hirta antagonizes gamma radiation induced radiotoxicity in lymphocytes in vitro. Complement. Integr. Med..

[16] Yu J., Zhu J., Chen L., Chao Y., Zhu W., Liu Z. (2023). A review of adsorption materials and their application of 3D printing technology in the separation process. Chem. Eng. J..

[17] Zheng Y., Li Q., Zhang G., Zhao Y., Liu X. (2021). Evaluation of separation effect for CH_4_ enrichment from coalbed methane (CBM) under the synergistic action of temperature and pressure based on IAST theory. GHG Sci. Technol..

[18] Chen Y., Wang T., Liu J., Huang J., Zhou G., Hu S. (2023). Synthesis of microgel-reinforced double network hydrogel adsorbent and its adsorption on heavy metals. J. Chem. Technol. Biotechnol..

[19] Li Y., Zhang Z., Liu B., Liu J. (2024). Adsorption of DNA oligonucleotides by boronic acid-functionalized hydrogel nanoparticles. Langmuir.

[20] Wang Z.J., Fu L.L., Liu D.L., Tang D.X., Liu K., Rao L., Yang J.Y., Liu Y., Li Y.S., Chen H.Q. (2023). Controllable preparation and research progress of photosensitive antibacterial complex hydrogels. Gels.

[21] Yang J.Y., Tang D.X., Liu D.L., Liu K., Yang X.J., Li Y.S., Liu Y. (2023). Excellent dark/light dual-Mode photoresponsive activities based on g-C3N4/CMCh/PVA nanocomposite hydrogel using electron beam radiation method. Molecules.

[22] Chen G., Ren K., Wang H., Wu L., Wang D., Xu L. (2023). Ga^3+^ loaded radiation crosslinked gel-Alg-CMC hydrogels for promoting diabetic wound healing. J. Biomater. Appl..

[23] Tang D.X., Liu K., Yang J.Y., Wang Z.J., Fu L.L., Yang X.J., Li Y.S., Huang B., Liu Y. (2024). Artificial nonenzymatic antioxidant Prussian blue/KGM-BSA nanocomposite hydrogel dressing as ROS scavenging for diabetic wound healing. Int. J. Biol. Macromol..

[24] Ansari M.J., Jasim S.A., Bokov D.O., Thangavelu L., Yasin G., Khalaji A.D. (2022). Preparation of new bio-based chitosan/Fe_2_O_3_/NiFe_2_O_4_ as an efficient removal of methyl green from aqueous solution. Int. J. Biol. Macromol..

[25] Bandura L., Panek R., Madej J., Franus W. (2021). Synthesis of zeolite-carbon composites using high-carbon fly ash and their adsorption abilities towards petroleum substances. Fuel.

[26] Jung K.W., Lee S.Y., Choi J.W., Hwang M.J., Shim W.G. (2021). Synthesis of Mg-Al layered double hydroxides-functionalized hydrochar composite via an in situ one-pot hydrothermal method for arsenate and phosphate removal: Structural characterization and adsorption performance. Chem. Eng. J..

[27] Rostami N., Faridghiasi F., Ghebleh A., Noei H., Samadzadeh M., Gomari M.M., Smith B.R. (2023). Design, synthesis, and comparison of PLA-PEG-PLA and PEG-PLA-PEG copolymers for curcumin delivery to cancer cells. Polymers.

[28] Zhou W., Hu Z., Wei J., Dai H., Chen Y., Liu S., Guo R. (2022). Quantum dots-hydrogel composites for biomedical applications. Chin. Chem. Lett..

[29] Yang J.Y., Liu K., Wang Z.J., Rao L., Fu L.L., Tang D.X., Yang X.J., Li Y.S., Chen H.Q., Liu Y. (2024). Electron beam radiation constructed and improved bone repairing performance based on hierarchical thermoplastic polyurethane/graphene oxide/hydroxyapatite hot melt adhesive with high flow ductility. Polym. Compos..

[30] Demeter M., Meltzer V., Călina I., Scărișoreanu A., Micutz M., Kaya M.G.A. (2020). Highly elastic superabsorbent collagen/PVP/PAA/PEO hydrogels crosslinked via e-beam radiation. Rad. Phys. Chem..

[31] Torkaman R., Maleki F., Gholami M., Torab-Mostaedi M., Asadollahzadeh M. (2021). Assessing the radiation-induced graft polymeric adsorbents with emphasis on heavy metals removing: A systematic literature review. J. Water Process. Eng..

[32] Lahsmin Y.K., Heryanto H., Ilyas S., Fahri A.N., Abdullah B., Tahir D. (2021). Optical properties determined from infrared spectroscopy and structural properties from diffraction spectroscopy of composites Fe/CNs/PVA for electromagnetic wave absorption. Opt. Mater..

[33] Singh W.I., Sinha S., Devi N.A., Nongthombam S., Laha S., Swain B.P. (2021). Investigation of chemical bonding and electronic network of rGO/PANI/PVA electrospun nanofiber. Polym. Bull..

[34] Li S., Luo C., Tang F., Xiao W., Fang M., Sun J., Chen W. (2022). Effect of polyethylene glycol modified MWCNTs-OH on the crystallization of PLLA and its stereocomplex. J. Polym. Res..

[35] Furer V.L., Vandyukov A.E., Tripathi V., Majoral J.P., Caminade A.M., Kovalenko V.I. (2017). Vibrational spectroscopic study on polycationic phosphorus dendrimers. Vib. Spectrosc..

[36] Sanjay V., Rajashekara K.M., Pattar V., Murugendrappa M.V. (2022). Effect on electrical and dielectric properties of Te nanoparticle-doped PVA composite. J. Mater. Sci. Mater. Electron..

[37] Yang Y., Kong W., Cai X. (2021). Synthesis and characterization of comb-like crosslinking polyurethane based form-stable phase-change materials for thermal energy storage. Polym. Advan. Technol..

[38] Tang R., Zhang Y., Zhang Y., Yu Z. (2016). Synthesis and characterization of chitosan based dye containing quaternary ammonium group. Carbohyd. Polym..

[39] Ezzati R., Azizi M., Ezzati S. (2024). A theoretical approach for evaluating the contributions of pseudo-first-order and pseudo-second-order kinetics models in the Langmuir rate equation. Vacuum.

[40] Moussavi S.P., Kadier A., Zaidi N.S., Aryanti P.T.P., Nugroho F.A., Wang J., Ma P.C. (2022). Removal of phosphorus from aqueous solution using multi-wall carbon nanotube (MWCNT) as adsorbent: Kinetics and isotherms. Fuller. Nanotub. Carbon Nanostruct..

[41] Miglioranza G., Schwaab M. (2023). Generalization and evaluation of the analytical solution of intraparticle diffusion models in finite batch adsorption. Macromol. React. Eng..

[42] Ojeme V.C., Ayodele O., Oluwasina O.O., Okoronkwo E.A. (2019). Adsorption of Pb (II) ions from aqueous solutions using chemically treated and untreated cow dung ash. BioResources.

[43] Sen T.K. (2023). Adsorptive removal of dye (Methylene blue) organic pollutant from water by pine tree leaf biomass adsorbent. Processes.

[44] Zou M., Zhang H., Miyamoto N., Kano N., Okawa H. (2022). Adsorption of an anionic surfactant (sodium dodecyl sulfate) from an aqueous solution by modified cellulose with quaternary ammonium. Polymers.

[45] Pan X.H., Zu J.H., Diao J.J., Xue Y., Liu S.Y. (2022). Rapid and selective recovery of Ag (I) from simulative electroplating effluents by sulfydryl-rich covalent organic framework (COF-SH) with high adsorption capacity. Colloids Surf. A Physicochem. Eng. Asp..

[46] Zeng L., Zhou F., Hu R., Xie W., Wang G., Yang C., Van der Bruggen B. (2021). Synthesis of cross-linked carboxyl modified polyvinyl alcohol and its application in selective adsorption separation of Cu (II) from Cd (II) and Ni (II). J. Polym. Environ..

[47] Dai S., Ye R., Huang J., Wang B., Xie Z., Ou X., Tian B. (2022). Distinct lipid membrane interaction and uptake of differentially charged nanoplastics in bacteria. J. Nanobiotechnol..

[48] Yan Y., Zhang C., Deng X., Zhang J., Xue Y., Zhang J., Chen J. (2023). Designing Superhydrophilic Hydrogels as Binder-free catalysts for enhanced oxygen evolution performance. Ind. Eng. Chem. Res..

[49] Murugan E., Akshata C.R., Ilangovan R., Mohan M. (2022). Evaluation of quaternization effect on chitosan-HAP composite for bone tissue engineering application. Colloids Surf. B Biointerfaces.

[50] Huang P., Zhang Y., Wang B., Zhu X., He Y., Song P., Wang R. (2023). Synthesis of acryloyl copolymer core-shell microspheres with antibacterial activity and surface cationic effects. J. Mater. Sci..

[51] Logigan C.L., Delaite C., Popa M., Băcăiță E.S., Tiron C.E., Peptu C., Peptu C.A. (2024). Poly (ethylene glycol) methyl ether acrylate-grafted chitosan-based micro-and nanoparticles as a drug delivery system for antibiotics. Polymers.

[52] Guo L., Hu K., Wang H. (2023). Antimicrobial and mechanical properties of Ag@Ti_3_C_2_Tx-modified PVA composite hydrogels enhanced with quaternary ammonium chitosan. Polymers.

